# Pig tongue soft robot mimicking intrinsic tongue muscle structure

**DOI:** 10.3389/frobt.2024.1511422

**Published:** 2025-01-09

**Authors:** Yuta Ishikawa, Hiroyuki Nabae, Megu Gunji, Gen Endo, Koichi Suzumori

**Affiliations:** ^1^ School of Engineering, Institute of Science Tokyo, Tokyo, Japan; ^2^ Department of Life Sciences, Faculty of Life Sciences, Toyo University, Tokyo, Japan

**Keywords:** soft robot, biomimetic robot, pig tongue, muscular structure mimicking robot, pneumatic artificial muscle

## Abstract

Animal muscles have complex, three-dimensional structures with fibers oriented in various directions. The tongue, in particular, features a highly intricate muscular system composed of four intrinsic muscles and several types of extrinsic muscles, enabling flexible and diverse movements essential for feeding, swallowing, and speech production. Replicating these structures could lead to the development of multifunctional manipulators and advanced platforms for studying muscle-motion relationships. In this study, we developed a pig tongue soft robot that focuses on replicating the intrinsic muscles using thin McKibben artificial muscles, silicone rubber, and gel. We began by performing three-dimensional scans and sectional observations in the coronal and sagittal planes to examine the arrangement and orientation of the intrinsic muscles in the actual pig tongue. Additionally, we used the diffusible iodine-based contrast-enhanced computed tomography (Dice-CT) technique to observe the three-dimensional flow of muscle pathways. Based on these observations, we constructed a three-dimensional model and molded the pig tongue shape with silicone rubber and gel, embedding artificial muscles into the robot body. We conducted experiments to assess both the motion of the tongue robot’s tip and its stiffness during muscle contractions. The results confirmed characteristic tongue motions, such as tip extension, flexion, and lateral bending, as well as stiffness changes during actuation, suggesting the potential for this soft robot to serve as a platform for academic and engineering studies.

## 1 Introduction

Soft robots can adapt to the external environment by taking advantage of their flexible bodies. They have been studied for a wide range of applications, such as versatile manipulators that can grasp a variety of objects without damaging them, and robots that can change shape and explore narrow space (Mazzolai et al., 2019; Sinatra et al., 2019; Shepherd et al., 2011; Hawkes et al., 2017). The high degree of freedom of soft robots is also suitable for reproducing the flexible movements of animals, and previous studies have mimicked the movements and bodies of various kinds of animals such as snake body, elephant trunk, octopus feet, and giraffe and ostrich necks (McMahan et al., 2006; Niikura et al., 2022; Nakano et al., 2023; Ma et al., 2023; Leanza et al., 2024).

Recent research has increasingly focused on robots that not only replicate external movements but also emulate the internal musculoskeletal structures found in biological organisms (Niikura et al., 2022; Nakano et al., 2023; Diamond et al., 2012; Asano et al., 2016; Richter et al., 2016; Kurumaya et al., 2016). By imitating these biological structures, robots are expected to realize soft, efficient, and smooth movements like animals. Furthermore, these biomimetic robots are expected to serve as a platform for investigating the relationship between movement and structural configuration of their bodies, and also as a platform that can provide an environment similar to that inside an actual organism for culturing biological tissues (Nakano et al., 2023; Mouthuy et al., 2022).

However, most studies on biomimetic robots with musculoskeletal structures have treated their muscles as if they were simple wires, i.e., focusing only on their tension-generating function and neglecting the physical volume changes of the muscles. Animal body is composed of soft tissues, including muscles, fat, and fascia, seamlessly layered from superficial to deep regions. This dense structure leads to close interaction between muscles resulting in changing the muscle configuration such as the position and direction of muscle pathways (Murai et al., 2016). In addition to these geometric characteristics, mechanical properties, such as changes in stiffness during muscle contraction, may affect their movements.

One particularly interesting organ that has a dense distribution of muscles, which operate by interfering with each other, is the tongue. Tongue is an important manipulator for animals that handle objects in vital behavior for living things, such as feeding and swallowing, and many researchers have studied its mechanisms (Cheng et al., 2002; Hiiemae and Palmer, 2003; Youmans and Stierwalt, 2006; Kennedy et al., 2010). Tongue is driven by four types of intrinsic tongue muscles and a larger variety of extrinsic tongue muscles, which are arranged three-dimensionally with intersecting muscle fibers in each muscle (Saito and Itoh, 2003; Sanders and Mu, 2013). Since the shape of the tongue has a significant role in its function in handling objects, the effects of changes in muscle volume are likely to be more significant. In previous studies, to reproduce the motion of the human tongue, several human tongue robots have been developed using pneumatic pressure, wires, and link mechanisms (Fukui et al., 2009; Lu et al., 2017; Shijo et al., 2019; Krisdityawan et al., 2023; Hofe and Moore, 2008). Among them, there was a robot that imitated the structure of the human tongue muscles by using wire-driven systems and reproduced the tongue behavior during human’s speech production (Hofe and Moore, 2008). However, these robots were designed to mimic the lingual behavior of humans, thus imitating the tongue muscle characteristics such as the dense structure, interfered configurations, or the mechanical properties, were not considered.

In this study, we developed a tongue-like soft robot that mimics the muscular structure of a tongue. The purpose of this study is to reproduce the flexible movement of the tongue by mimicking the three-dimensional (3D) structure of the tongue muscles, and to explore its potential as a soft robotic manipulator as well as a platform for academic and engineering development. This paper focuses on 1) reproducing the intrinsic muscular structure of tongue to realize both the 2) basic motion and 3) mechanical characteristics (variable stiffness) of a tongue, the latter of which has not been explored at depth in robotic tongues. Although there are six basic spatial motions–three translational motions and three rotational motions–the translational motions, comprising protrusion, retraction, elevation, depression, and bilateral motion, are mostly realized by the extrinsic muscles of the tongue (Kayalioglu et al., 2007; McClung and Goldberg, 2000). Moreover, among the rotational motions, it is unclear which muscles are the main contributors to twisting motion, even in the field of biology (Ross et al., 2024). Therefore, in this paper, we define the two bending motions, flexion-extension and lateral bending, as the basic movements of the intrinsic tongue muscles. Considering availability, we used a pig tongue as the target and observed the structure of its intrinsic muscles to make a 3D model of it. Based on this model, we fabricated a tongue-like soft robot. We measured its motion and stiffness during muscle activity and discussed the potential applications of the robot.

## 2 Materials and methods

### 2.1 Study of the arrangement of pig tongue muscles

The tongue muscles consist of two major muscle groups: 1) intrinsic muscles, which structure the tongue body, and 2) extrinsic muscles, which connect the tongue body to the external bone, or fascia. The intrinsic tongue muscles are classified into four types: Superior Longitudinal (SL), Inferior Longitudinal (IL), Transverse (T), and Vertical (V), according to the direction and position of the muscle pathways. The intrinsic tongue muscles are considered to contribute to the translation of the tip of the tongue and to the deformation of the shape of its body. On the other hand, the external tongue muscles are considered to be involved in large movements of the entire tongue.

These muscles are arranged three-dimensionally in various directions. In particular, the intrinsic muscles cross over each other, so it is not easy to identify their locations. In previous anatomical studies, researchers employed mainly two methods: histological methods, where cross-sectioned specimens were observed with the naked eye or under a microscope, and tomographic methods, where 3D images were obtained using imaging techniques such as computed tomography (CT) and magnetic resonance imaging (MRI) (KIER and SMITH, 2008; Abd-El-Malek, 1939; Thomas et al., 2013; Voskuilen et al., 2019). Histological techniques allow for detailed observation of the tissue structure, such as muscle fiber orientation, arrangement, and intersection with each fiber, however it is difficult to observe the 3D connections between the sections of the specimen. On the other hand, tomographic methods, while limited in observing fine details due to resolution constraints, are useful for examining the general distribution of the internal structure of specimen without damaging it. In recent years, the diffuse iodine-based contrast-enhanced CT (Dice-CT) method, which allows visualization of non-skeletal soft tissues via diffuse iodine staining, has become commonplace (Gignac et al., 2016). This is a powerful tool for understanding the three-dimensional structures of soft tissues that were previously difficult to image via CT scan.

In this study, two methods were utilized: 1) a cross section of the tongue, heated with boiling water to improve the visibility of the muscles (hereafter “heating method”), used to observe the muscle arrangement at each section, and 2) Dice-CT, used to observe the overall muscle arrangement.

#### 2.1.1 Heating method

Empirically, heat-induced discoloration of muscle is widely known. However, we have not seen it used as a method for anatomical investigation, particularly that of muscle structures. In this method, muscle fibers are observed by discoloring them with hot water poured over a tongue section. Although the intensity of discoloration depends on the temperature of the hot water, in this study, we decided to use boiling water (approximately 100°C) based on the results of a pre-experiment where we observed discoloration of muscle fibers in chicken breast fillet. Combined with observations from Dice-CT (explained later), we found the heating method to be easier than other histological approaches and sufficient for the purposes of this paper.

**FIGURE 1:**
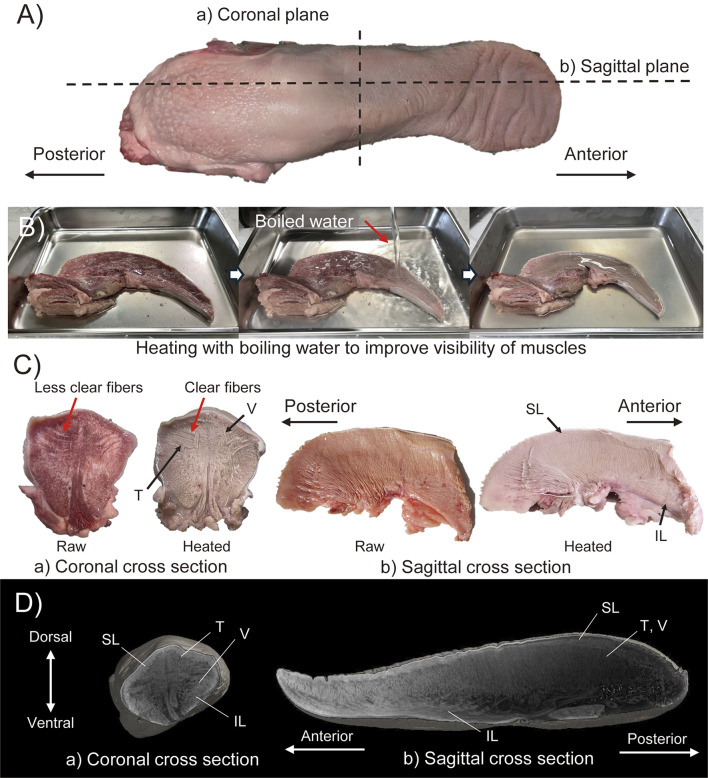
Muscle structure observation with **(A–C)** heating method and **(D)** Dice-CT method. **(A)** Raw material indicating the cut line of cross sectioned materials in heating method. **(B)** Images during heating method. **(C)** Results of heating method with coronal and sagittal cross section specimens. **(D)** CT-images of coronal and sagittal cross section obtained by Dice-CT method.

Figure 1A shows the appearance of the raw pig tongue. Figure 1B shows the process of this technique, and Figure 1C shows the raw and heated sections of each of the coronal and sagittal planes (as labeled in Figure 1A). In order to observe the intrinsic muscles, two tongues were used to make each section. Note that the tip of the tongue is omitted in the sagittal plane section shown in Figure 1C. From the heated section, the intersection of T and V muscle fibers can be clearly observed in the coronal plane (see red arrows in Figures 1A–C). It can also be seen that the T muscles are concentrated in half of the dorsal region in the coronal plane. In the sagittal plane, the V muscles pass perpendicular to the dorsal surface. In addition, the SL and IL muscles run longitudinally just under the dorsal surface and the anterior part of the ventral surface epithelial layers, respectively. In both sections, it is difficult to distinguish muscles running perpendicular to the cross-sectional plane.

#### 2.1.2 Dice-CT method

The Dice-CT method is a contrast method that allows nondestructive observation of both hard and soft tissues via CT, where the diffusible iodine spread throughout the soft tissues is revealed as contrast in the CT image (Gignac et al., 2016). In this study, pig tongues were stained with a 1 % w/v iodine-ethanol solution for 2 weeks and then destained with 99 % ethanol for 6 days. The results are shown in Figure 1D. Note that the lower part of the pig tongue where extrinsic muscles were attached was removed. From the figures, it can be confirmed that the T muscle is concentrated on the dorsal surface of the tongue, consistent with the results of the heating method. Compared to the heating method, the longitudinal tongue muscle located just below the epithelium is more clearly visible and we are able to follow its 3D path (not just the 2D cross-section) from its origin to its insertion point.

### 2.2 3D model for pig tongue robot

#### 2.2.1 Body of the pig tongue robot

**FIGURE 2:**
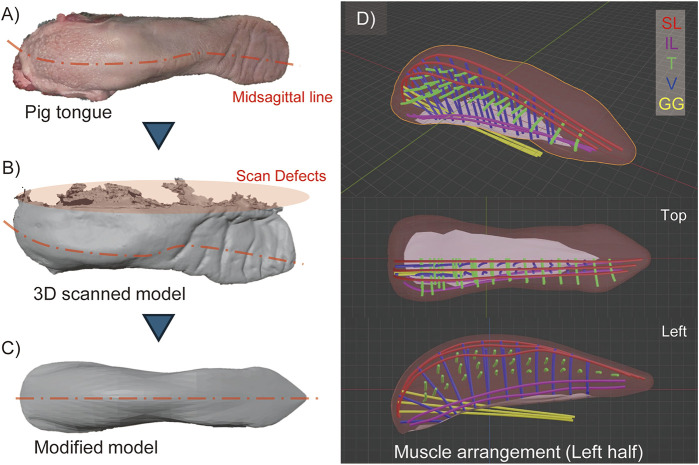
3D model of pig tongue. **(A)** Raw pig tongue indicating the deformation of its midsagittal line, **(B)** 3D scanned model, **(C)** Modified model of pig tongue where the model is deformed as the midsagittal line is straightened and mirrored in lateral direction, **(D)** The arrangement of the intrinsic muscles (SL, IL, T, and V muscles) and one kind of extrinsic tongue muscle (GG).

To obtain the actual shape of the tongue, a 3D model of a pig tongue was acquired using a 3D capture device (Revopoint Inc., Revopoint mini) and associated software (Revopoint Inc., Revo Scan 5). Due to the tongue’s softness, it was deformed by gravity while resting on the measurement stage, resulting in an asymmetrical shape. Additionally, the bottom surface in contact with the stage was hidden and not measurable, leading to a defect in the 3D model. To address these issues, we corrected the missing data and deformation by modifying the model using Blender, a 3D modeling software, to straighten the midline of the tongue and create a symmetrical model by mirroring the right side of the scanned model. Figures 2A–C, illustrates the model modification process.

#### 2.2.2 Arrangement of tongue muscles in 3D model

Based on the 3D model of the tongue and observations of the muscle structure, we determined the positions of the intrinsic tongue muscles and one extrinsic muscle, the genioglossus muscle, which originates from the back of the chin and inserts into the posterior region of the tongue. Figure 2D illustrates the arrangement of these muscles. Although this study does not primarily focus on extrinsic muscles, we included the genioglossus muscle because it occupies a significant part of the tongue body. In Figure 2D, each muscle is depicted as a 3 mm diameter curve to represent the size of the pneumatic artificial muscles, which will be described later. The tongue model is designed symmetrically, with both the left and right sides featuring three SL, two IL, twenty-seven T, and twenty-one V muscles, respectively. While the actual muscles in the pig tongue are densely braided, the muscles in our model are spaced apart to avoid obstructing the radial expansion of the pneumatic artificial muscles.

### 2.3 Manufacturing the pig tongue robot

The pig tongue robot is fabricated according to the tongue model. The general workflow for fabrication is as follows.

1. Based on the model, a mold and cores for making the epithelium of the tongue robot are fabricated.
2. The epithelium itself is fabricated via silicone rubber molding process.
3. Artificial muscles are placed at the aforementioned positions of the intrinsic tongue muscles.
4. The inside of the robot body is filled with silicone gel.

**FIGURE 3:**
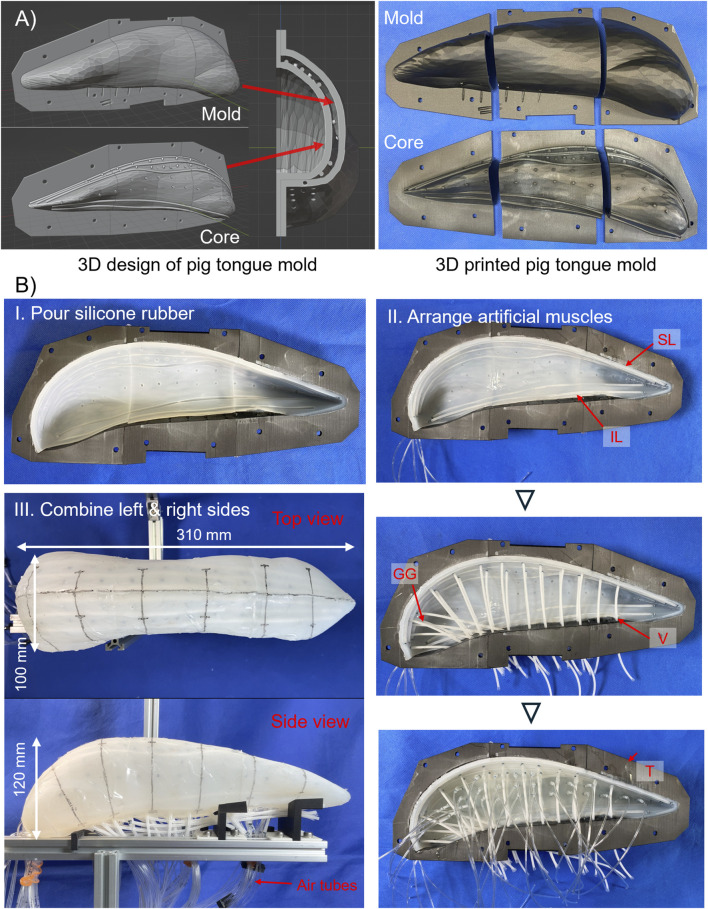
Making process of pig tongue robot. **(A)** Design of mold for pig tongue. Left shows 3D model of the mold and right shows 3D-printed mold and core. **(B)** I. Appearance of molded skin layer of the tongue robot, and II. actual arrangements of thin McKibben artificial muscles. III. Left- and right-side robot were combined, and air tube were installed.

The silicone gel fills the inside of the tongue to fix the position of each muscle while maintaining the flexibility of the entire tongue body. In addition, filling the volume of the robot with silicone gel serves to mimic the muscular hydrostat nature of a tongue (Kier and Smith, 2008). Figure 3 shows the rough procedure of the robot fabrication, from the 3D model to the assembled pig tongue soft robot. Note that the pig tongue model was scaled by 150% to account for the size of the artificial muscles, resulting in length, width, and height dimensions of approximately 310, 100, and 120 mm, respectively.

#### 2.3.1 3D-printed mold for tongue robot

First, to fabricate the skin layer of the robot, the mold is designed in 3D modeling software. The mold for the tongue robot is created based on the model obtained from the 3D scan. The overview of the mold is shown in Figure 3A.

The mold for the tongue robot mainly consists of two parts: an outer mold for the overall shape of the tongue and an internal mold (core) that defines the shape of the epithelial part of the robot. By pouring liquid silicone rubber into the space between the outer mold and the core, the epithelium layer can be fabricated. This core determines the thickness of the epithelium and the location of the intrinsic tongue muscles. On the surface of the core, we design convex ridges which make concave grooves on the inner surface of the epithelium layer, and temporarily fix the artificial muscles to these grooves during the fabrication process. Since the artificial muscles have a radius of 1.5 mm, the thickness of the epithelium layer was set to 3.0 mm. In addition, some holes are drilled at the bottom of the mold to pipe the air tubes required for driving the pneumatic artificial muscles used in this study.

#### 2.3.2 Thin McKibben artificial muscle

The muscles of the tongue are arranged three-dimensionally and run in various directions. As a result, when the tongue deforms, the muscles are subjected to various loads, including tension, bending, and torsion. Consequently, artificial muscles must be both durable enough to withstand these loads and flexible enough to allow for natural deformation. In this study, thin McKibben artificial muscles are used as actuators (Kurumaya et al., 2017). These pneumatically driven linear actuators are durable up to 0.5 MPa, with a diameter of 2 or 3 mm, and their high flexibility allows them to be used as fibers in fabrics and textiles (Kurumaya et al., 2017; Funabora, 2017; Kilic Afsar et al., 2021; Hiramitsu et al., 2023). Although they require an air supply tube due to being air pressure-driven, they do not require a motor, offering greater design flexibility in actuator placement compared to wire-based systems.

Though there are a total of 106 artificial muscles in our tongue robot, the hydrostat mechanism of a biological tongue is considered to be accurately replicated since the proportion of the artificial muscles’ volume on the whole structure is approximately 5.5% (also note that, for the following experiments, only certain muscle groups were activated at a time).

#### 2.3.3 Molding process

For the molding process, two types of elastomers are used: silicone rubber, which is used for epithelium layer, and silicone gel, which is used for filling the inside of the robot. First, a core is placed inside the mold, and silicone rubber (Smooth-On, Ecoflex 00–10) is poured into the space between the mold and core and is cured to form the epithelial part of the tongue robot. The mold is divided into three parts, anterior, middle, and posterior, which enables the silicone rubber to be poured from the cross sections that separate each part, and facilitate removal of the core after the rubber has cured. After the core is removed, the same silicone rubber is applied to the cross-section surface of each part to bond them together.

After bonding, the artificial muscles are placed in the robot. The muscles are positioned along the concave patterns, which were imprinted by the core on the inner surface of the epithelium layer. To ensure that the artificial muscles remain in place and do not shift during assembly, a small amount of the same silicone rubber used for the epithelial part is applied to the contact points between the muscles and epithelium layer. After the muscles are arranged, the inner space of the tongue robot is filled with silicone gel (Smooth-On, Ecoflex GEL). The same process is repeated for both the left and right sides of the tongue. After attaching the air tubes to the artificial muscles corresponding to the T muscles of the tongue robot, they are routed to the exterior through the ventral side of the robot, and the left and right tongue are bonded together with silicone gel. Finally, the exposed gel surface is covered with the silicone rubber, and the remaining artificial muscles are fitted along with air supply tubes, completing the pig tongue robot.

## 3 Experiments

In this section, we describe the experimental method. Due to the high number of muscles, it is not realistic to check the movement of each muscle by driving them one by one. In this study, therefore, the tongue is considered to be divided into three regions: anterior, middle, and posterior, and thus, the internal tongue muscles are divided into SL, IL, and anterior, middle, and posterior transverse ( $T_{a}$ , $T_{m}$ , and $T_{p}$ ) and vertical ( $V_{a}$ , $V_{m}$ , and $V_{p}$ ) muscle groups.

On the surface of the robot, a mid-sagittal line and lateral lines at approximately 50 mm intervals from the anterior to the posterior of the tongue are marked to enhance the visibility of deformation.

With this configuration we measured the motion, deformation, and change in stiffness of the pig tongue robot.

### 3.1 Movement of intrinsic tongue muscles

With the muscle classification described above, the tongue robot was actuated, and two measurements were taken: 1) the displacement of the tongue tip, and 2) the deformation of the lateral and vertical dimensions during the activation of each muscle group.

First, the displacement of the tongue tip was measured using motion tracking in the xyz directions, with cameras facing the top (1980 × 1,080, 30 fps) and lateral sides (3860 × 2160, 60 fps). To ensure consistency between both views, using Blender (a 3D modeling and video editing software), tracking points were set at both the tongue tip and root in each view, and the pixel distance between the points was understood as the length of the robot: 310 mm.

**FIGURE 4:**
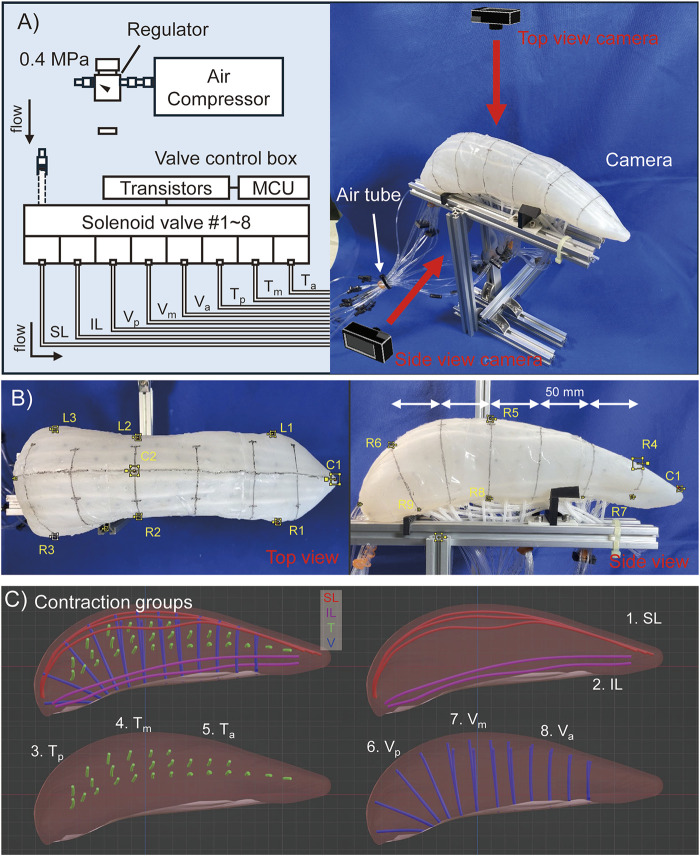
Experimental configuration of pig tongue motion tracking. **(A)** Rough image of setting of the pig tongue robot. **(B)** Motion tracking marker point on the robot images. **(C)** Contraction groups of the muscles which are simultaneously activated in a motion.

Second, to assess tongue deformation, the width and thickness of the tongue were measured in the anterior, middle, and posterior regions. During motion tracking, the tip of the tongue was designated as point C1, and three points on the right and left contour lines of the robot were labeled as $R_{1}$ , $R_{2}$ , $R_{3}$ , $L_{1}$ , $L_{2}$ , and $L_{3}$ , respectively, from anterior to posterior. Additionally, in the lateral view, motion tracking markers were placed along the lines drawn on the tongue surface: $R_{4}$ , $R_{5}$ , and $R_{6}$ on the dorsal surface line, and $R_{7}$ , $R_{8}$ , and $R_{9}$ on the ventral surface line of the tongue robot. Tongue width changes were determined from the length changes between ( $R_{1}$ , $L_{1}$ ), ( $R_{2}$ , $L_{2}$ ) and ( $R_{3}$ , $L_{3}$ ), while thickness changes were derived from the length changes between ( $R_{4}$ , $R_{7}$ ), ( $R_{5}$ , $R_{8}$ ), and ( $R_{6}$ , $R_{9}$ ). Any errors that arose during application of the automatic tracking function in Blender were manually corrected. Figure 4 provides an overview of the measurement setup and the locations of the motion tracking markers on the tongue robot. Note that the robot is not fixed to the base (which would prevent contraction of muscles near the robot’s surface), but simply laid on jigs to prevent rotation.

### 3.2 Stiffness measurement of pig tongue soft robot

The stiffness of a biological tongue is varied through the contraction of muscles; in other words the longitudinal elastic modulus of the tongue increases during muscle contraction (Shibata et al., 2012). Therefore, in this study, to measure the mechanical properties of the tongue robot, the elastic force when the dorsal surface of the tongue was compressed was measured under four states: 1) during deactivation, 2) when the T muscles were driven, 3) when the V muscles were driven, and 4) when both were driven. The measurement position on the dorsal tongue was set at point $C_{2}$ on the midline of the tongue (shown in Figure 4B). In this measurement, a force gauge (NIDEC, FGP-50) attached to the linear stage was pressed against the tongue robot, sandwiching it against the aluminum frame to obtain the relationship between displacement and force. A circular probe with a diameter of 11 mm was attached to the tip of the force gauge.

The detail of the measurement procedure is as follows.

1. A force gauge attached on the linear stage is moved in 1 mm increments toward the robot, and the position where 1 N of compression force is generated is set as the reference position.
2. The force gauge is moved toward the robot in 5 mm increments, and the force measured every step until it reaches 50 mm.
3. Repeat the above processes four times.
4. Change the muscle states and repeat them.

The stage was always moved at a maximum speed of 2 mm/s.

## 4 Results

### 4.1 Movement of tongue tip during intrinsic muscle contraction

**FIGURE 5:**
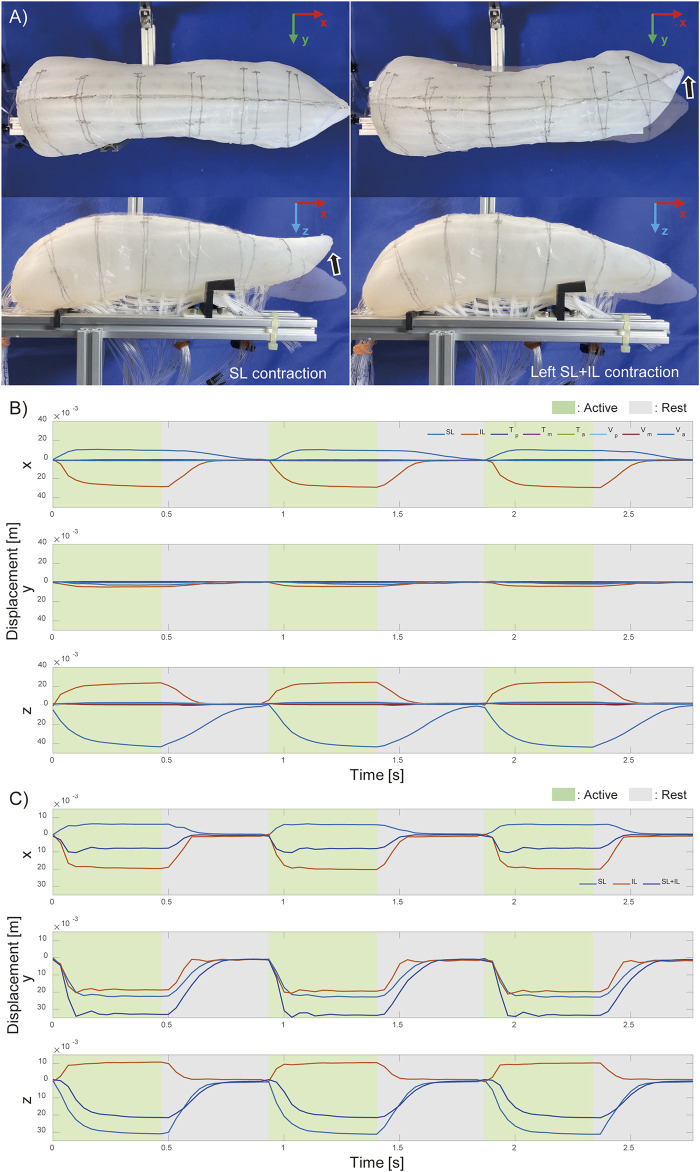
Motion of pig tongue soft robot. **(A)** Actual motion of the pig tongue with SL and IL contraction, and Motion tracking results of the tip of the tongue robot **(B)** when each intrinsic muscles contracts and **(C)** when left side of longitudinal muscles contract.

Supplementary materials S1, S2 demonstrate the movement of the tongue robot during each muscle group contraction. The most pronounced movements were observed during the contraction of the SL and IL muscles, as shown in Figure 5A. The xyz coordinate system is oriented such that the x -axis is positive from the posterior to the anterior regions of the tongue, the y -axis is positive from the right side to the left side of the tongue, and the z -axis is positive in the upward direction. In Figure 5B, the initial position of the tongue is set at 0, and the muscle contractions are performed in the following order: SL, IL, $T_{p},T_{m},T_{a},V_{p},V_{m}$ , and $V_{a}$ . From Figure 5B, the largest movements of the tongue tip are observed during the contraction of the SL and IL muscles across all xyz directions. In contrast, the V and T muscle groups show minimal movement in the x - and z -directions. Since the robot was designed symmetrically, displacement along the y -axis was almost zero. However, the SL and IL muscles tended to move more laterally compared to the other muscles during contraction. This could be due to asymmetry caused by human error during the manual manufacturing process and accidental roll of the robot on the workbench.

Figure 5C illustrates the movement of the tongue tip when the longest muscles (SL, IL, and both together) were contracted, on the left side only. By activating SL and IL on just one side, left-right vertical oblique movements were achieved. When SL and IL were contracted simultaneously, lateral bending movements were observed with minimal displacement in the vertical direction. The results shown in Figure 5C correspond to the contractions of the muscles on the left side.

Figure 5 demonstrates that, even using only the intrinsic tongue muscles, it is possible to control fundamental movements, such as anterior-posterior and lateral flexion of the tongue tip, by driving SL and IL in different directions.

**FIGURE 6:**
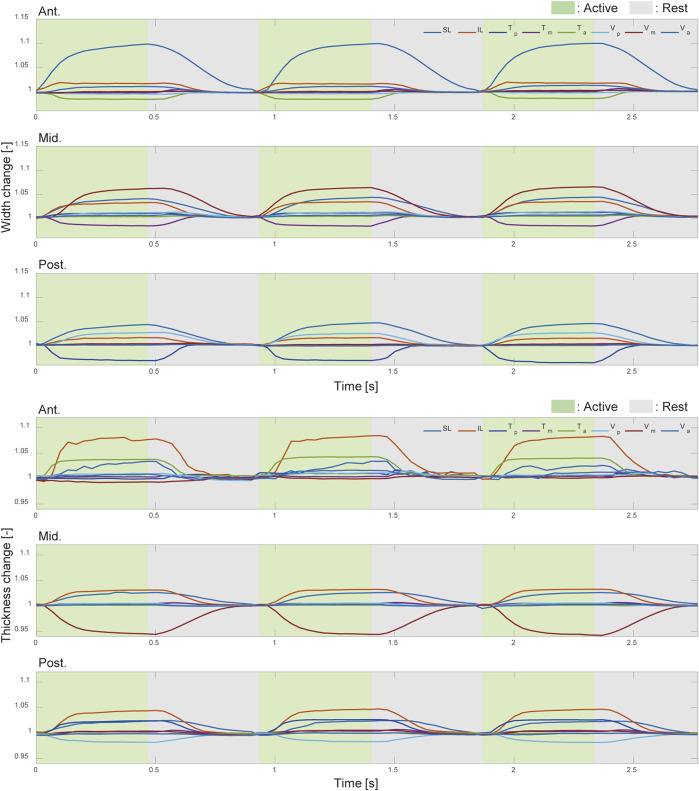
Width and thickness change with intrinsic muscle contraction. The deformation ratio of the width (top) and thickness (bottom) in the anterior, middle, and posterior regions of the pig tongue robot. Green and gray regions indicated the corresponding muscle was active and inactive, respectively.

Figure 6 shows the results of measuring changes in the width and thickness across three regions of the tongue robot during contraction of each muscle group. The graphs illustrate the changes in width and thickness at the anterior, middle, and posterior regions of the tongue from left to right. These changes are expressed as ratios relative to the initial length of each measurement point at the start of muscle contraction.

From Figure 6, the width and thickness changes at the anterior and middle parts are affected by the contraction of the muscles present in those regions. Interestingly, at the posterior and the anterior regions of the tongue robot, the reduction in width and thickness with muscle contraction is limited to a maximum of 2 % , whereas in the direction of expansion, the ratio is relatively large, reaching a maximum of 10 % . It can be seen that the displacement perpendicular to the tongue pathways is larger than that in the parallel direction. On the other hand, in the middle region, both of these contractions and expansions were less than 6 % in magnitude. These results suggest that the effects of the T and V muscles differ depending on the longitudinal section of the tongue.

### 4.2 Stiffness changes with intrinsic tongue muscles

**FIGURE 7:**
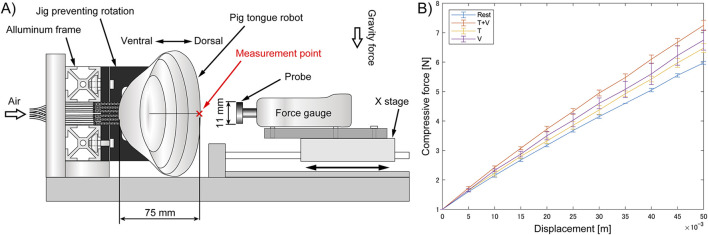
Relationship between the displacement and compressive force against middle part of the pig tongue robot, **(A)** Experimental setup, **(B)** Result.

Changes in the stiffness of the dorsal tongue were measured with and without contraction of the T and V muscles. The results are shown in Figure 7, where measurements were taken four times under each state and the average is shown in the graph.

The force-displacement relationship of the pig tongue robot during compression was nearly linear across the measurement range, regardless of muscle contraction. The data indicated an increase in tongue stiffness during the contraction of the V and T muscles compared to when the muscles were not activated. Furthermore, contracting the vertical and transverse tongue muscles simultaneously resulted in a greater increase in stiffness compared to when each muscle was contracted individually. When the force gauge was pressed 50 mm against the robot surface, the elastic force averaged 5.98 N at rest and 7.25 N during contraction of the T and V muscles, indicating a stiffness increase of approximately 1.2 times. Considering the dimensions of the robot (thickness of 75 mm, measurement area of 95.0 mm^2^), the Young’s moduli of the tongue with and without the muscle contractions were 94 kPa and 114 kPa, respectively.

## 5 Discussion

### 5.1 Comparison with biological and conventional robotic tongues

In this study, we replicate the structure of intrinsic tongue muscles. Although the amount of the robot’s motion is relatively small compared to a biological pig tongue (due to the lack of extrinsic tongue muscles), the two basic bending motions, the flexion-extension and lateral bending motions, were realized. Also, the shape deformation function was realized by replicating the intrinsic tongue muscles.

To evaluate the performance of our robot, it is compared against measurements from a biological pig tongue and conventional robots. Since the motion range of a biological tongue is mainly determined by the extrinsic tongue muscles and jaw movement, it is difficult to compare it with our robot. On the other hand, the deformation ratios in width and thickness induced by intrinsic muscles are suitable for evaluating the performance of our robot; these values are compared in Table 1. Table 2 compares the range of motion of our robot with conventional tongue-like robots.

**TABLE 1:** Shape deformation ratio of biological and proposed tongue.

	Width change (%)		Thickness change (%)	
	Ant.	Post.	Ant.	Post.
Pig tongue^a^	20–33	10–20	—	15–25
Proposed robot	−2–10	−4–4	5–10	5–7.5

^a^ Shcherbatyy and Liu (2007).

**TABLE 2:** Motion of conventional robotic tongue.

	Length (mm)	Displacement (mm)
Robotic tongue^a^	40	20
Licker^b^	85	80^c^
WT-6^d^	75^e^	20^f^
The proposed robot	310	60

^a^ Lu et al. (2017).

^b^ Shijo et al. (2019).

^c^ Deformation in the upper and lower direction, estimated from the figure.

^d^ Fukui et al. (2007), Fukui et al. (2009).

^e^ Estimated from the figure.

^f^ Including the deformation of jaw.

The width and height change of tongue are considered to be mainly induced by intrinsic tongue muscles such as transverse and vertical muscles. It was reported that the deformation ratio of the width of a pig tongue during chewing was approximately 10%–20% in the posterior region and 20%–33% in the anterior region, and the ratio of the thickness was 15%–25% in the posterior region (Shcherbatyy and Liu, 2007). For our robot, the deformation ratios of the posterior width and thickness ranged from approximately −4 to 4% and −2–4.5%, respectively. Thus, the total range of posterior deformation is 6%–8%, which gives us a performance of approximately half the range of dimensional deformation of a biological pig tongue. Conversely, the deformation ratio of the anterior width of the tongue was less than a half of that of the biological pig tongue.

A possible explanation for this relatively small deformation ratio is the lack of the muscles near the tip of the tongue combined with the comparative hardness of the silicone rubber. Presently, it is difficult to manufacture thin pneumatic muscles that are compact enough to fit in the small area at the tip of the tongue. Further, the hardness of the silicone rubber used as the epithelium layer of the robot is about 55 kPa at 100% modulus. This is also insufficient for matching the softness of a biological pig tongue, which is less than 10 kPa (Dresselhuis et al., 2008). To realize a more flexible soft robot, we must consider these factors as issues in future work.

### 5.2 Comparison of the variable stiffness

The stiffness of our tongue robot was 94 kPa (as a Young’s modulus) at rest, and increased to 114 kPa upon contraction of the T and V muscles. This is higher than the Young’s modulus of the silicon rubber alone, and around 10 times higher than that of a biological pig tongue, which is less than 10 kPa (Dresselhuis et al., 2008). This may be due to the relatively higher stiffness of the thin McKibben artificial muscles compared to the silicon materials used in the body.

Further, when the T and V muscles were contracted simultaneously, stiffness measurements under compressive force only increased by 1.2 times (compared to the at-rest values). In contrast to the six-fold change in elastic modulus observed in human tongues during muscle activity (Shibata et al., 2012), we speculate that the relatively smaller increase in our tongue robot may be the result of low muscle density.

## 6 Conclusion

In this paper, we developed and evaluated a soft robot designed to mimic the complex, three-dimensional muscular structure of a pig tongue. The robot’s tongue tip movements were assessed at various states, including contraction of individual muscle groups and muscles on one side. We demonstrated that basic tongue movements, such as flexion-extension and lateral bending, can be achieved through the combined action of SL and IL muscles, and by activating muscles on the left and right sides.

Measurements of width and thickness changes in the tongue’s three regions (anterior, middle, and posterior) indicated that while muscle contractions affected these dimensions, they did not significantly alter the tip’s movement. This limited effect could be attributed to the stiffness of the tongue’s surface and the constraints imposed by non-driven muscles.

Stiffness measurements in response to compressive force resulted in a 1.2-fold increase when the T and V muscles were contracted simultaneously, compared to the resting state. This is relatively small compared to the six-fold change in elastic modulus observed in human tongues with muscle activity (Shibata et al., 2012). Potential reasons for this discrepancy include 1) the absence of external tongue muscles, 2) differences in muscle density, and 3) variations in properties between artificial and biological muscles. Although the thin diameter of artificial muscles allows for soft bending, the inherent stiffness of the nylon fibers becomes noticeable when embedded in silicone rubber or similar materials, as observed in this robot.

In conclusion, this study emulated the muscular structure of a pig tongue using thin McKibben artificial muscles and mimicked characteristics such as basic tongue movements, deformations, and changes in stiffness. While the study focused solely on the intrinsic muscles of the tongue, the actual biological tongue also includes extrinsic muscles that work with the intrinsic ones simultaneously. Although the displacement, deformation at the tip of the tongue robot, and stiffness changes in the middle region of the robot are smaller compared to actual biological tongues, the validity of these results should be discussed in the context of the interaction with extrinsic muscles. Additionally, the overall stiffness of the tongue robot seems to be greater than that of a biological tongue, indicating a need for improved flexibility.

While we can qualitatively show that certain milestones have been achieved, quantitatively, future work should aim to develop a more realistic robot by enhancing flexibility and incorporating the extrinsic tongue muscles. This will require exploring other materials (e.g., rubber) and artificial muscles, as well as investigating muscle density and arrangement. Also, we think the structure of the innervation of the biological tongue is a good reference for reproducing the tongue function of living organisms in the robot, both in terms of motor control and sensing. Considering such innervation structure, embedding soft sensors and soft touch sensors that do not compromise the flexibility of the robotic tongue could be a useful platform for understanding the function of such organisms.

## Data Availability

The original contributions presented in the study are included in the article/Supplementary Material, further inquiries can be directed to the corresponding author.
